# Supplementary material for: Optimization of Extraction Methods for NMR and LC-MS Metabolite Fingerprint Profiling of Botanical Ingredients in Food and Natural Health Products (NHPs)
Source: Molecules. 2025 Aug 14;30(16):3379. doi: 10.3390/molecules30163379 (PMC12388471; doi:10.3390/molecules30163379)
Supplement: Supplementary file 1 [file molecules-30-03379-s001.zip › molecules-3746699-supplementary.pdf]

Optimization of Extraction Methods for NMR and LC-MS Metabolite Fingerprint Profiling of Botanical Ingredients in Food and Natural Health Products (NHPs)

Varathan Vinayagam [1,†], Arunachalam Thirugnanasambandam [1,2,*,†], Subramanyam Ragupathy [1], Ragupathy Sneha [1,3] and Steven G. Newmaster [1]

1 College of Biological Science, University of Guelph, Guelph, ON N1G 2W1, Canada; vinaychemist89@gmail.com (V.V.); ragu@uoguelph.ca (S.R.); snehara@auamed.net (R.S.); snewmast@uoguelph.ca (S.G.N.)

2 Biological and Life Sciences, Canadian Light Source, Saskatoon, SK S7N 2V3, Canada

3 College of Medicine, American University of Antigua, Jobberwock Beach Road, Coolidge P.O. Box W1451, Antigua and Barbuda

* Correspondence: athiru@outlook.com

† These authors contributed equally to this work.

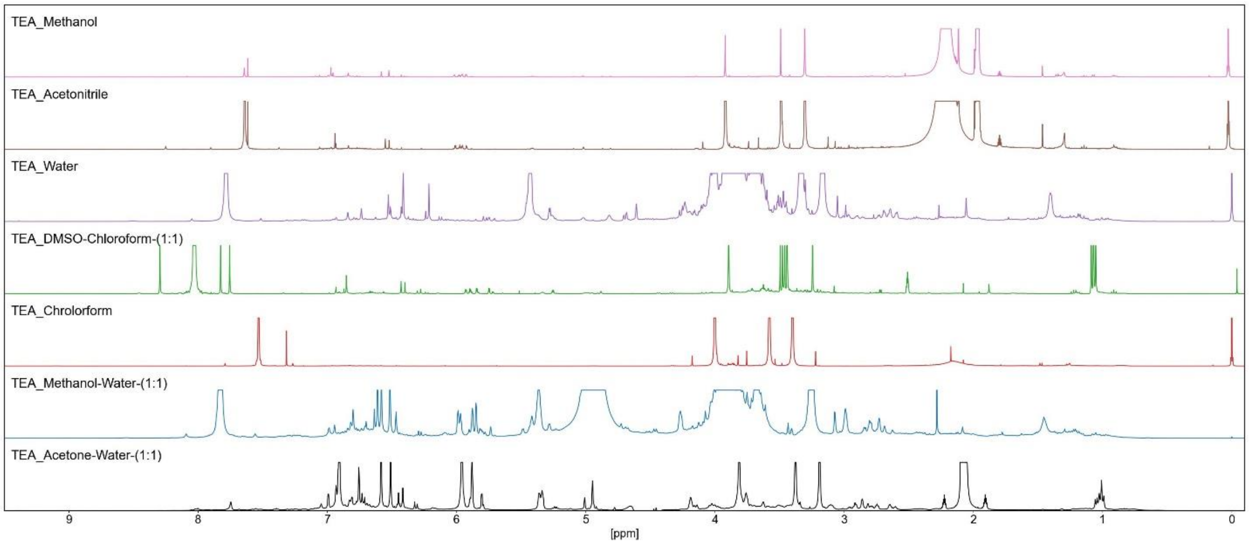

Figure S1. ^{1}H NMR spectra (400 MHz) of *Camellia sinensis* (tea) extracts obtained using different solvents and solvent mixtures. Spectra are displayed from top to bottom: methanol (pink), acetonitrile (brown), water (purple), DMSO-chloroform (1:1, green),

chloroform (red), methanol-water (1:1, blue), acetone-water (1:1, cyan). Chemical shifts are reported in δ (ppm) relative to TMS.

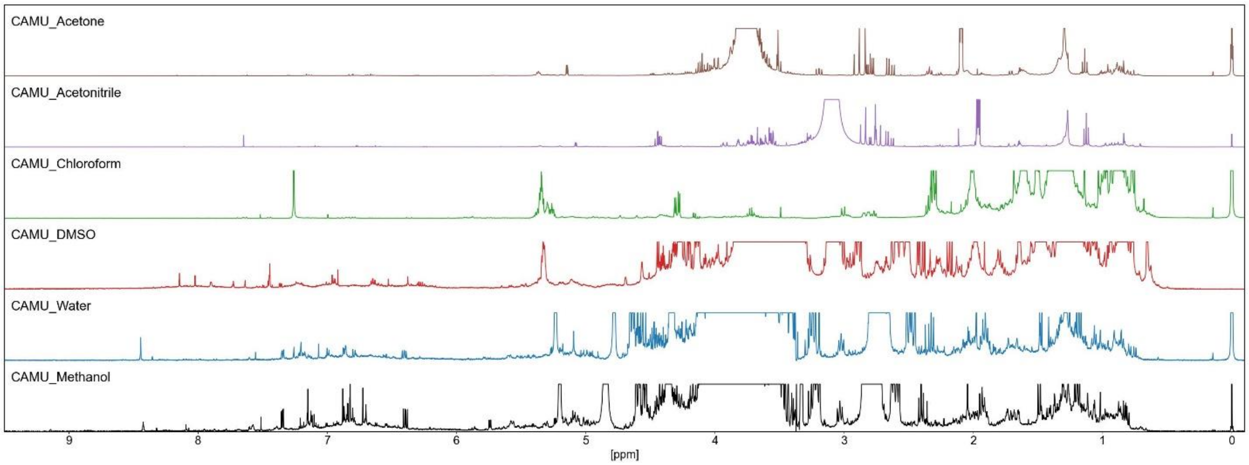

Figure S2. ^{1}H NMR spectra (400 MHz) of *Myrciaria dubia* (camu camu) extracts obtained using different solvents. Spectra are displayed from top to bottom: acetone (brown), acetonitrile (purple), chloroform (green), DMSO (red), water (blue), methanol (black). Chemical shifts are reported in δ (ppm) relative to TMS.

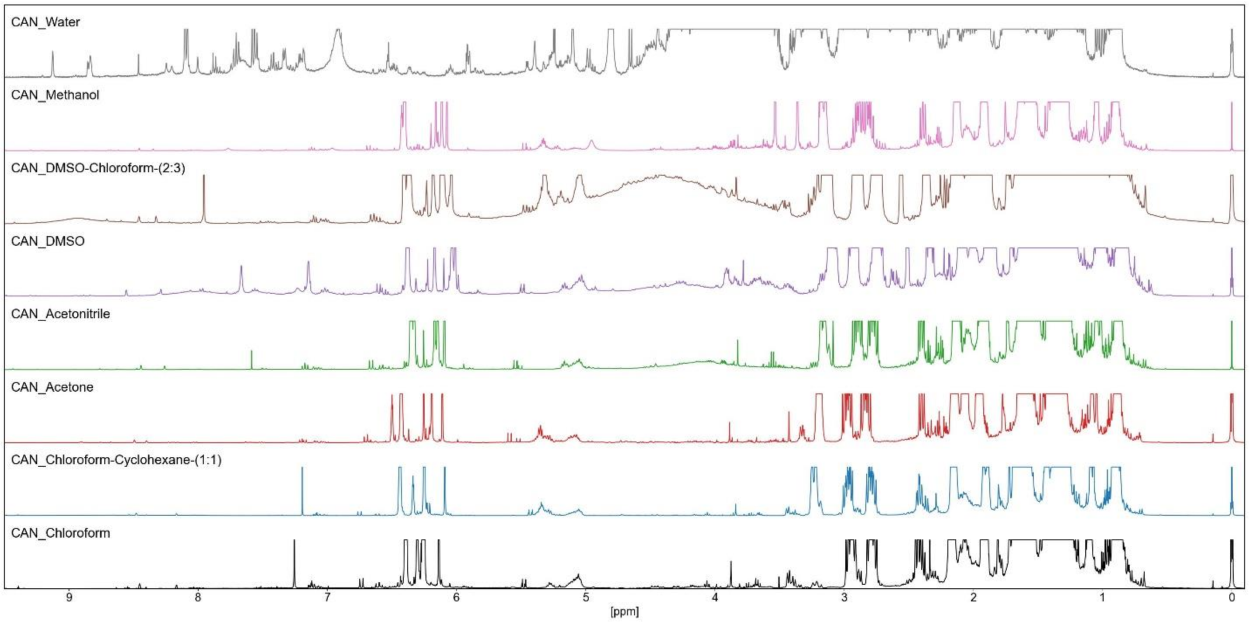

Figure S3. ^{1}H NMR spectra (400 MHz) of *Cannabis sativa* extracts obtained using different solvents and solvent mixtures. Spectra are displayed from top to bottom: water (black), methanol (pink), DMSO-chloroform (2:3, brown), DMSO (purple), acetonitrile (blue), acetone (green), chloroform-cyclohexane (1:1, red), chloroform (blue). Chemical shifts are reported in δ (ppm) relative to TMS.

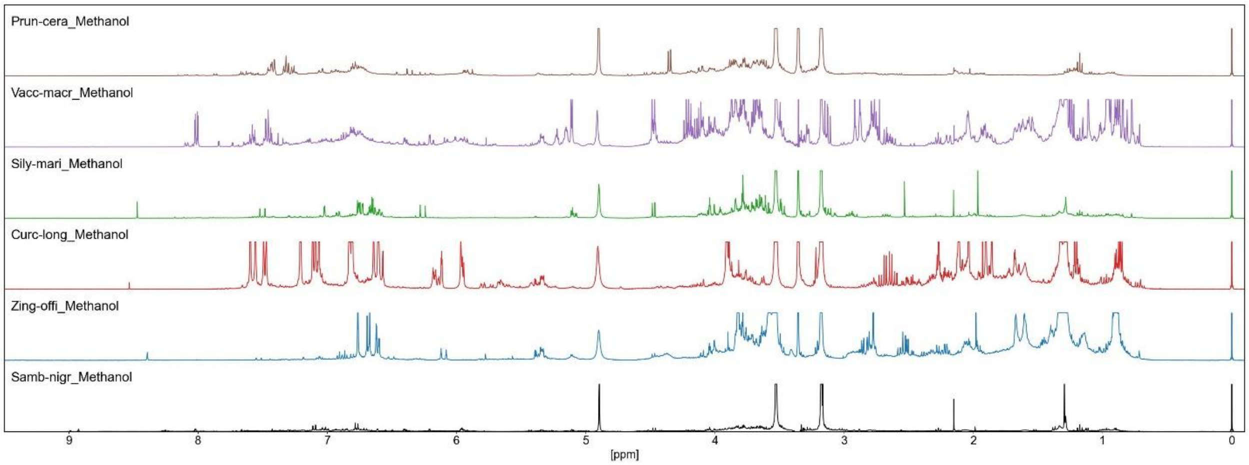

Figure S4. ^{1}H NMR spectra (400 MHz) of various botanical extracts in methanol solvent. Spectra are displayed from top to bottom: *Prunus cerasus* (brown), *Vaccinium macrocarpon* (purple), *Silybum marianum* (green), *Curcuma longa* (red), *Zingiber officinale* (blue), *Sambucus nigra* (black). Chemical shifts are reported in δ (ppm) relative to TMS.

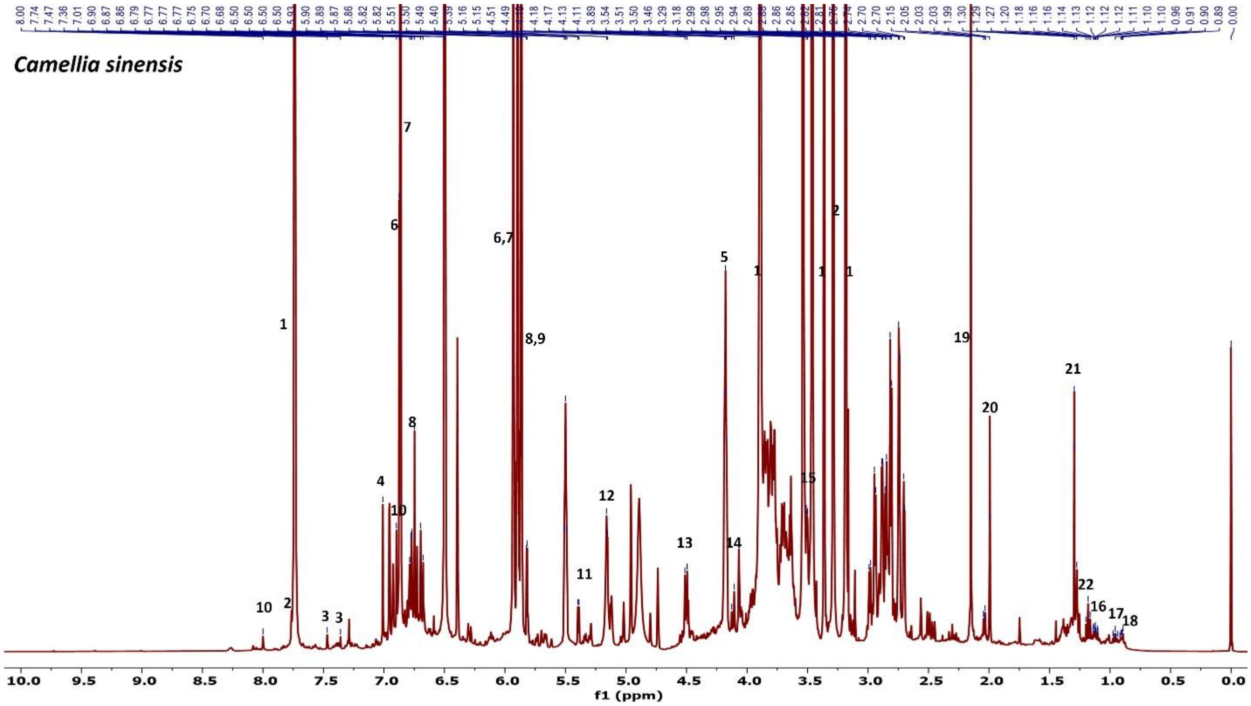

Figure S5. Assigned ^{1}H NMR spectrum (400 MHz) of *Camellia sinensis* extract in methanol-d_4, with characteristic peaks labeled for key metabolites (refer to Table 4 for detailed assignments, multiplicities, and J-coupling constants). Chemical shifts are reported in δ (ppm) relative to TMS.

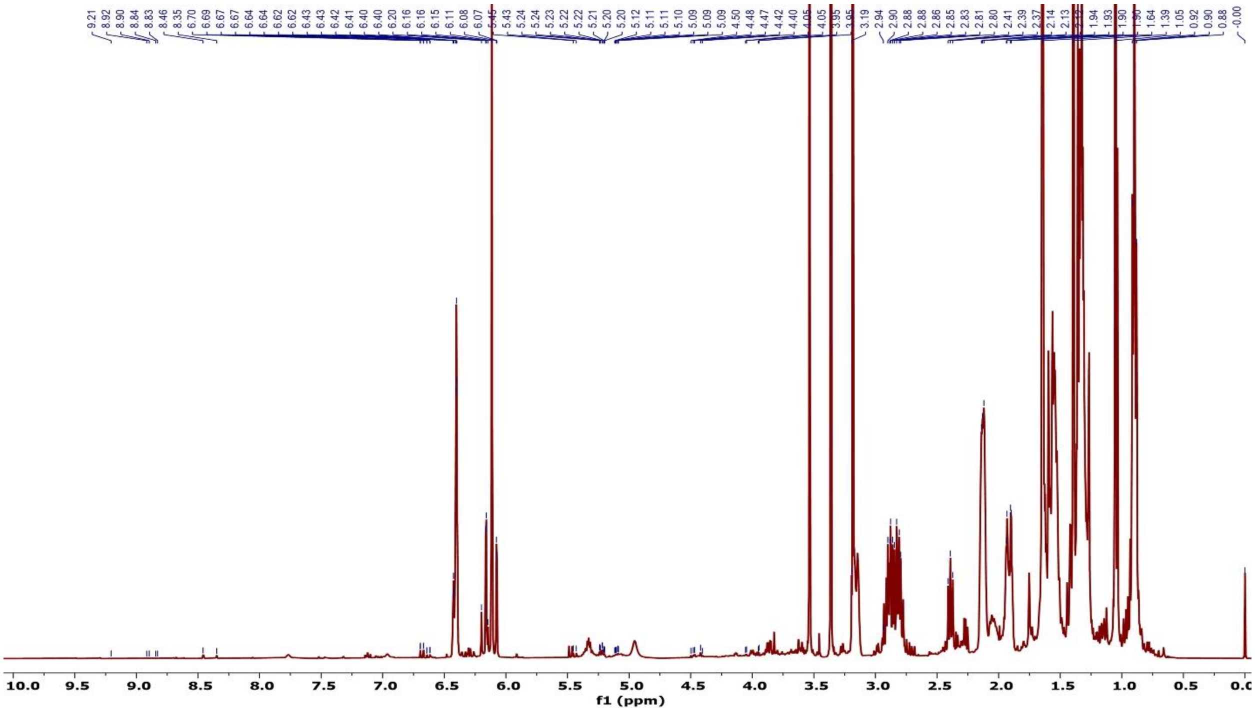

Figure S6. Assigned ^{1}H NMR spectrum (400 MHz) of *Cannabis sativa* extract in methanol-d_4, with characteristic peaks labeled for key metabolites (refer to Table 4 for detailed assignments, multiplicities, and J-coupling constants). Chemical shifts are reported in δ (ppm) relative to TMS.

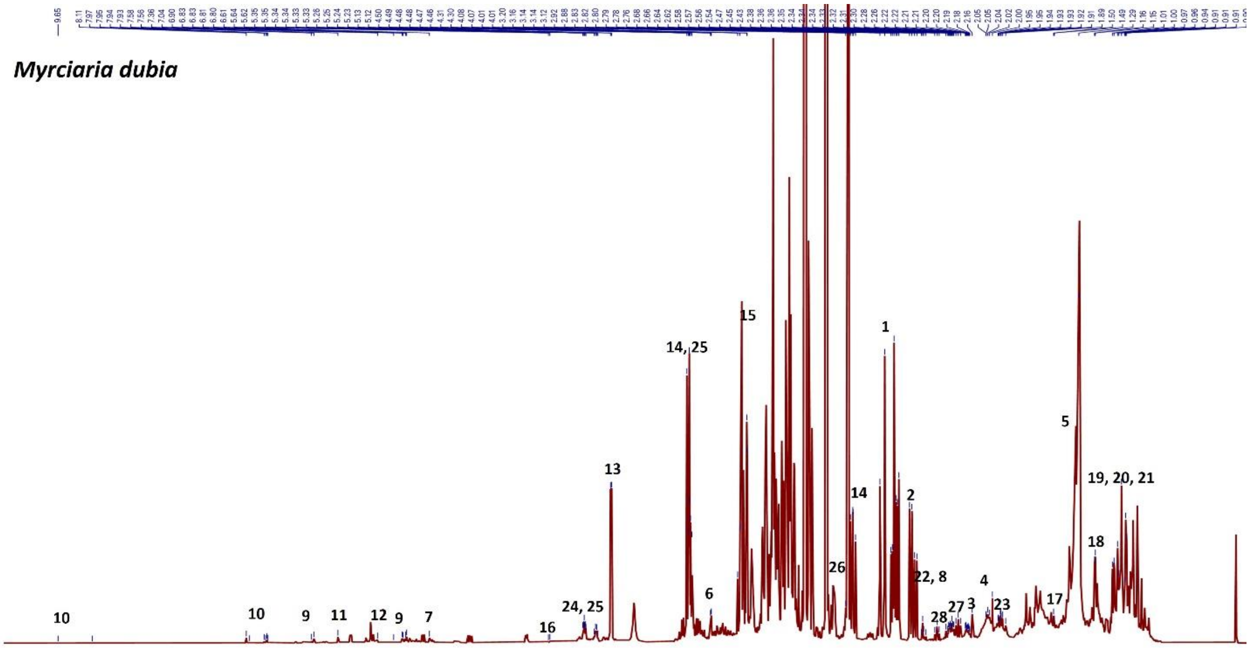

Figure S7. Assigned ^{1}H NMR spectrum (400 MHz) of *Myrciaria dubia* extract in methanol-d_4, with characteristic peaks labeled for key metabolites (refer to Table 4 for detailed

assignments, multiplicities, and J-coupling constants). Chemical shifts are reported in δ (ppm) relative to TMS.
